# Conformational Selection in Enzyme‐Catalyzed Depolymerization of Bio‐based Polyesters

**DOI:** 10.1002/cbic.202400456

**Published:** 2024-09-17

**Authors:** Ximena Lopez‐Lorenzo, Ganapathy Ranjani, Per‐Olof Syrén

**Affiliations:** ^1^ School of Engineering Sciences in Chemistry, Biotechnology and Health Department of Fibre and Polymer Technology KTH Royal Institute of Technology Stockholm 100 44 Sweden; ^2^ School of Engineering Sciences in Chemistry, Biotechnology and Health Science for Life Laboratory Tomtebodavägen 23 Solna 171 65 Sweden

**Keywords:** Biocatalysis, Bio-based polymers, Conformational selection, Enzymatic degradation

## Abstract

Enzymatic degradation of polymers holds promise for advancing towards a bio‐based economy. However, the bulky nature of polymers presents challenges in accessibility for biocatalysts, hindering depolymerization reactions. Beyond the impact of crystallinity, polymer chains can reside in different conformations affecting binding efficiency to the enzyme active site. We previously showed that the *gauche* and *trans* chain conformers associated with crystalline and amorphous regions of the synthetic polyethylene terephthalate (PET) display different affinity to PETase, thus affecting the depolymerization rate. However, structural‐function relationships for biopolymers remain poorly understood in biocatalysis. In this study, we explored the biodegradation of previously synthesized bio‐polyesters made from a rigid bicyclic chiral terpene‐based diol and copolymerized with various renewable diesters. Herein, four of those polyesters spanning from semi‐aromatic to aliphatic were subjected to enzymatic degradations in concert with induced‐fit docking (IFD) analyses. The monomer yield following enzymatic depolymerization by IsPETase S238 A, Dura and LCC ranged from 2 % to 17 % without any further pre‐treatment step. The degradation efficiency was found to correlate with the extent of matched substrate and enzyme conformations revealed by IFD, regardless of the actual reaction temperature employed. Our findings demonstrate the importance of conformational selection in enzymatic depolymerization of biopolymers. A straight or twisted conformation of the polymer chain is crucial in biocatalytic degradation by showing different affinities to enzyme ground‐state conformers. This work highlights the importance of considering the conformational match between the polymer and the enzyme to optimize the biocatalytic degradation efficiency of biopolymers, providing valuable insights for the development of sustainable bioprocesses.

## Graphical abstract

The significance of conformational selection in enzymatic degradation of biopolymers is demonstrated for a series of terpene‐derived polyesters. A fundamental 1D‐geometric parameter with respect to whether the polymer chain is straight or twisted impacts biocatalytic depolymerization by influencing the polymer's affinity to enzyme ground state conformers. Two representative enzyme residues in PETase are shown in blue and magenta.

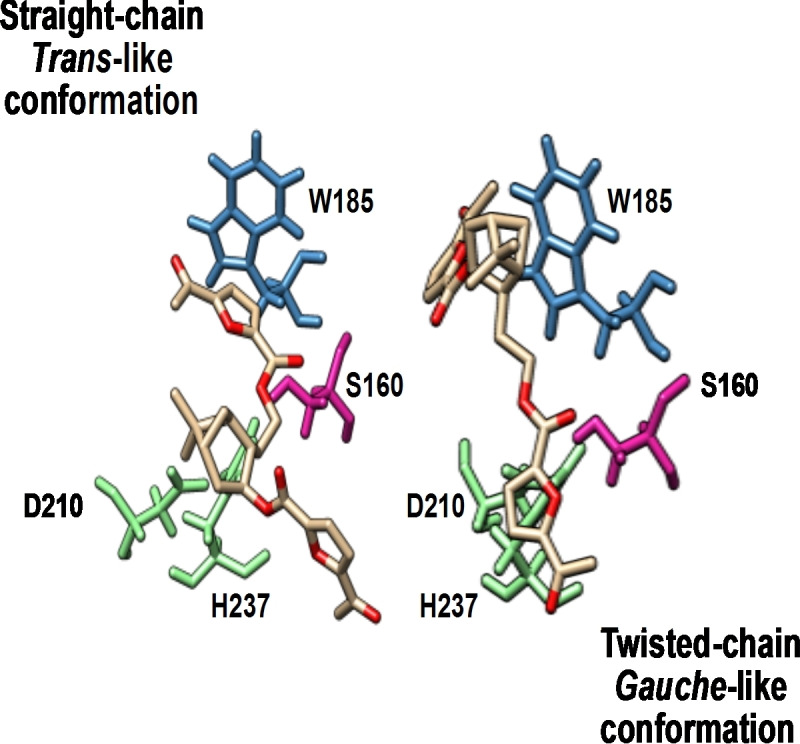

## Introduction

Synthetic polymers found in medical devices, textiles, computers, and many other applications have become vital for life. The overall polymer market is mainly composed of the following polymers: polyethylene (PE) 36 %, polypropylene (PP) 19 %, polyvinylchloride (PVC) 12 %, and polyester 21 %. The production of polymer fabrics is largely made up of polyethylene terephthalate (PET) (70 %).[^1^, ^2^] The production of polyesters in 2022 increased to 63 million tons in comparison to the 61 million tons produced in 2021. By 2030, global polyester fiber production is expected to grow up to 145 million tons.^[2]^ Polyesters are versatile materials that can be synthesized from a wide variety of monomers; including bio‐mass‐derived monomers.[^4^, ^5^] via various polymerization routes, including polycondensation and ring‐opening polymerization (ROP). A prominent example is polyfuranoates (PEF) in which the fossil ‐based terephthalic acid was replaced by the bio‐based monomer 2,5‐furan dicarboxylic acid (FDCA)).[^3^, ^4^]

Although polyesters contain hydrolyzable bonds, they can still withstand biodegradation.^[5]^ The generation of biodegradable and fully recyclable bio‐based polymers is necessary to achieve circular materials^[6]^ with mechanical and thermal properties reminiscent to fossil‐based materials. The most common recycling methods for polyesters are chemical and mechanical recycling^[7]^ and the combination of these approaches can effectively recycle polyesters, but at a great cost of energy and under harsh conditions.^[8]^ To circumvent these issues, focus has recently been on biocatalysts that can hydrolyze ester bonds under mild conditions.^[9]^ Employing water‐based systems for polymer recycling at mild temperatures is an alternative to the classical chemical and thermomechanical recycling routes. Since synthetic polyesters have been around for a relatively short time, there hasn't been enough time for nature to evolve existing enzymes to become efficient enough to replace mechanical and chemical recycling techniques.

In 2005, a cutinase from the organism *Thermobifida fusca* (TfH) was found to partially degrade PET.^[10]^ Moreover, in 2016, the *Ideonella sakaiensis* PETase (*Is*PETase) was isolated from a plastic bottle found in a plastic landfill and was shown to be able to use PET as its partial carbon source.^[11]^ These two enzymes have an evolutionary relationship by sharing ca. 51 % sequence identity and display the classical *α*/*β* ‐hydrolase fold.[^12^, ^13^] Even though their sequence identity is moderate, they share important structural characteristics as these two enzymes have in common that their active site is located on their surface, facilitating the bulky polymeric substrate to access the catalytic triad. Enzyme engineering strategies including rational design, directed evolution, and machine learning approaches[^14^, ^15^] have been used to obtain more thermostable variants of *Is*PETase, e. g. the Dura PETase variant that has a melting temperature (*T*_m_) of 80 °C^[16]^; allowing for degradation of PET at temperatures exceeding the glass‐transition temperature (*T*_g_), and resulting in increased monomer yields.

**Figure 1:**
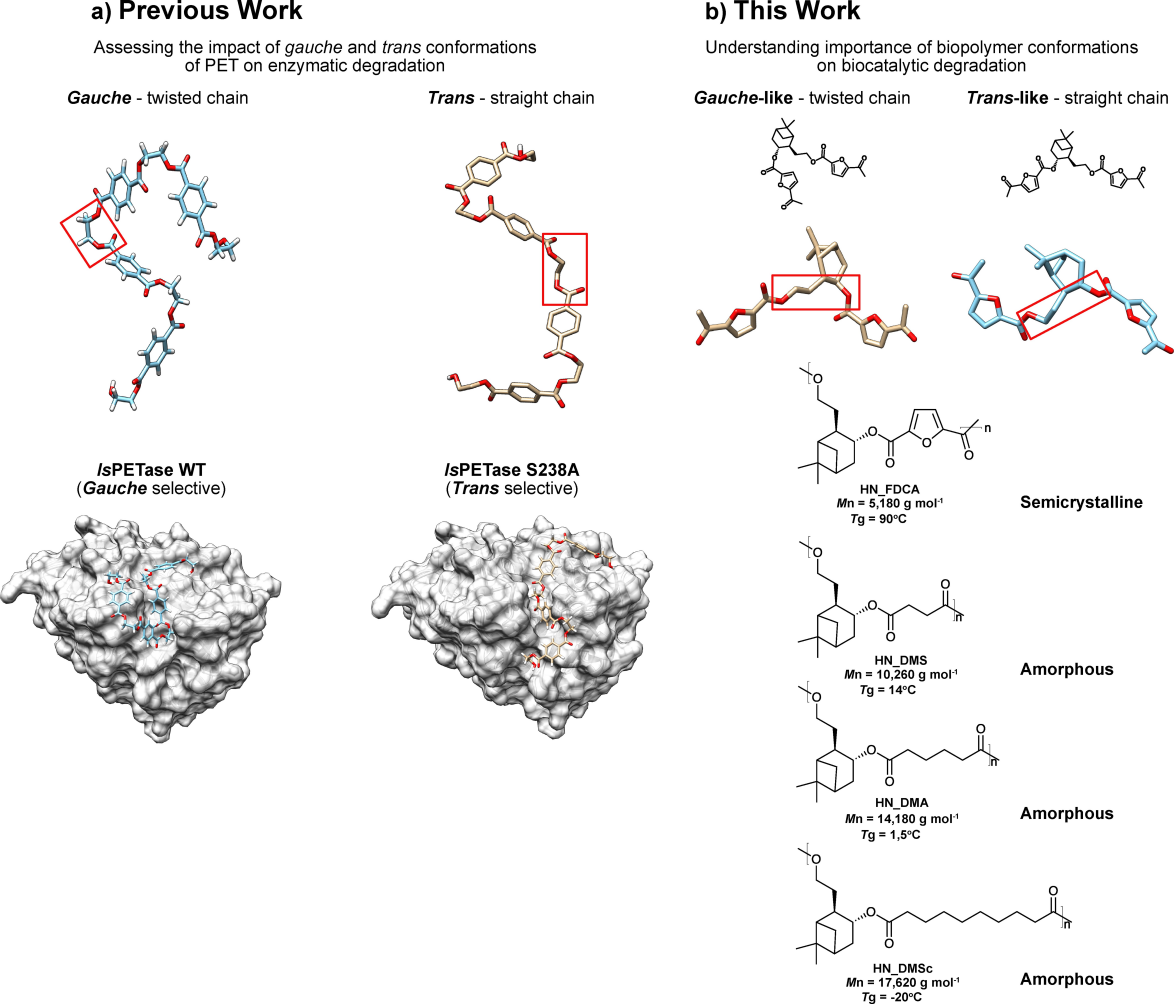
a) In our previous work we showed how the two PET substrate conformations *trans* and *gauche* will have an impact on the depolymerization efficiency through different binding affinity of these straight or twisted polymer chains to discrete enzyme variants. Figure adapted from Guo *et al*. (2022) and Guo *et al*. (2023) b) This work highlights the importance of the biopolymer conformation on enzymatic degradation efficiency. *Top*: Schematic drawing of how the terpene‐derived bio‐polyesters under study can adopt either a twisted chain (*gauche*‐like) or *trans*‐like structure (straight chain). *Bottom*: Biopolymers investigated in this work. HN_FDCA is a semi‐crystalline polyester and HN_DMS, HN_DMS, and HN_DMSc are amorphous polyesters. The molecular weight (*M*_n_ from SEC) and glass transition temperature (*T*_g_) of each polymer substrate is shown.

As a complementary strategy to improve enzyme thermostability, enzyme‐catalyzed depolymerization of polyesters can be improved by better understanding the enzyme and substrate conformations[^18^, ^19^] and their interplay with each other to form productive complexes prone to undergo catalysis. Hitherto structure‐functional relationship data has mainly focused on the impact of material crystallinity and molecular weight on the enzymatic depolymerization rate of synthetic polyesters.[^17^, ^18^] In our previous work, we showed the importance of conformational selection^20^ for *Is*PETase, its variants and LCC cutinase[^12^, ^19^] (Leaf and Branch Compost Cutinase) for efficient hydrolysis of PET (Figure 1a) into its constituent monomers.[^20^, ^21^] Still, the importance of biomolecular recognition of discrete polymer chain conformations remains poorly understood,^[20]^ especially for biopolymers.

For this study, we combined induced‐fit docking with enzyme‐catalyzed depolymerization studies to shed light on structure‐functional relationships underpinning the biodegradation of four bio‐based polyesters synthesized from a rigid pinene‐derived diol co‐polymerized with esters of FDCA and aliphatic bio‐mass derived diacids (Figure 1b).^[25]^ The nature of the four polymers investigated varied from crystalline aromatic to amorphous. NMR and SEC analysis revealed the ability of two *Is*PETase variants (S238 A and Dura PETase^[16]^) and a cutinase (LCC) to effectively depolymerize both crystalline and amorphous polyesters without any mechanical or chemical pre‐treatment step; irrespectively of the reaction temperature that was ambient for *Is*PETase S238 A (30 °C) and 70 °C for LCC cutinase and Dura PETase, respectively. The degradation efficiency of the enzymes was associated with their capabilities to harbor different polymer conformations, and particularly with respect to how the diol chains in the polymer unit are organized relative to one another (see Figure 1b, top) resulting in either straight or twisted chains.

## Results and Discussion

The four polyesters shown in Figure 1b were produced by transesterification followed by polycondensation of the terpene‐based diol called *trans*‐hydroxy nopol (herein referred to as, HN). The monomer HN was synthesized from (*R*)‐(−)‐Nopol which is a natural product and commercially available pinene‐based compound that can be synthesized from *α*‐pinene.^[22]^ Since *α*‐Pinene is inert, and cannot be used as such for polymer synthesis without first being subjected to functionalization, a retro‐synthetic route starting from (*R*)‐(−)‐Nopol was used^[21]^ involving hydroboration followed by oxidation to obtain HN (see methods). Using the bulky bicyclic diol (HN) in concert with the bio‐based dimethyl esters of 2,5‐furan dicarboxylic acid (FDCA), succinic acid (DMS), adipic acid (DMA), and sebacic acid (DMSc) resulted in one crystalline (HN_FDCA) polyester, and three amorphous aliphatic polyesters (HN_DMS, HN_DMA, HN_DMSc); respectively. Their molecular weights ranged from ca 5000–18000 g mol^−1^ (Figure 1b, bottom). Polymerization of the bulky rigid diol (HN) with the rigid aromatic diester (FDCA) resulted in a syndiotactic polymer that is highly crystalline, whereas the flexible aliphatic diesters reacted with the diol (HN) randomly to afford amorphous atactic polymers.^[21]^ While the difference in morphological properties confers different application possibilities, we hypothesized that any associated difference in the conformational landscape of the polymer chains could alter bio‐degradability by PETases. Accordingly, we focused on the structural relationship of these novel bio‐based polyesters towards enzymatic degradation.

### Enzymatic Depolymerization of Polyesters

The resulting terpene‐derived polyesters were shown to be susceptible to enzymatic depolymerization by all three enzymes investigated, two *Is*PETase variants (S238 A and Dura) and a cutinase (LCC). These enzymes have previously been shown by us to display a preference towards either *trans*‐rich (referring to the dihedral angle of the ethylene glycol unit, S238 A PETase) or *gauche*‐rich (Dura PETase and LCC cutinase) polyethylene terephthalate (PET)‐derived substrates.[^20^, ^21^] To study whether analogous chain conformations in biopolymers could affect the enzymatic rate, depolymerization reactions of the four polyesters shown in Figure 1b were performed at the optimal temperature for each enzyme for 72 hours. For S238 A PETase, the reaction was performed at 30 °C, and for Dura PETase and LCC cutinase at 70 °C.

**Figure 2:**
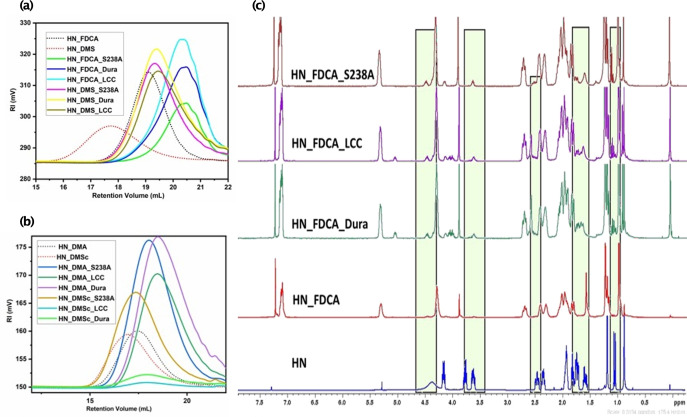
Characterization of the enzymatically depolymerized polyesters. The resulting degradation products were measured from a 72 hour enzymatic depolymerization reactions with S238 A PETase (30 °C), Dura PETase (70 °C) and LCC cutinase (70 °C). (a) Comparison of SEC data for the polyesters HN_FDCA and HN_DMS before and after enzymatic degradation. (b) Comparison of SEC data for the polyesters HN_DMA and HN_DMSc before and after enzymatic degradation. (c) Representative ^1^H NMR spectra showing monomer formation at *δ* 4.5 ppm following enzymatic hydrolysis of the polyester HN FDCA. See supporting information for spectra of other polymers.

Following the enzymatic reactions, the obtained polymeric products were first subjected to SEC analysis to confirm the hydrolysis of the polymer chain and to be able to determine the molecular weight (*M*_n_) of the resulting oligomers. The starting molecular weights of the polyesters were 5,180 g mol^−1,^ 10,260 g mol^−1^, 14,180 g mol^−1,^ and 17,620 g mol^−1^ for HN_FDCA, HN_DMS, HN_DMA and HN_DMSc respectively. The HN_FDCA polyester was the most swiftly degraded by S238 A PETase enzyme variant concerning the release of monomers (calculated from ^1^H NMR, see Figure 2c, Table 1). Also, the resulting oligomeric product (Figure 2a and 2b) had a *M*_n_ of 1,653 g mol^−1^ suggesting that the enzyme successfully degraded the polymer into shorter chain oligomers. Both the Dura PETase and LCC had a higher activity towards the amorphous aliphatic polyesters, and HN_DMSc polyester being the most susceptible one to degradation. The *M*_n_ obtained after enzymatic degradation was 1,649 g mol^−1^ and 1,614 g mol^−1^. The results obtained from SEC were further corroborated by NMR spectra of the polymer samples after enzymatic degradation to study the release of the monomers.

**Table 1:** Table summarizing the results following enzymatic degradation.

**Polyester^[a]^**	**Initial** ***Đ*** ^**[b]**^	**Yield of the monomer by NMR (%)^[c]^**			**Mn**_**(SEC)**_ **(g/mol)** ^**[d]**^			**Mw**_**(SEC)**_ **(g/mol)** ^**[e]**^			**After enzymatic degradation** ***Đ*** ^**[f]**^		
		**S2388 A**	**Dura**	**LCC**	**S238 A**	**Dura**	**LCC**	**S238 A**	**Dura**	**LCC**	**S238 A**	**Dura**	**LCC**
HN FDCA	1.3	17	7	7.5	1653	1604	1749	2642	2831	2954	1.60	1.77	1.69
HN DMS	1.7	6	7	8	2464	1782	1706	5085	4500	4259	2.06	2.50	2.56
HN DMA	1.6	3.5	13.4	10.0	4999	2669	2976	13805	8818	9256	2.76	3.30	3.11
HN DMSc	1.9	2.0	12.6	10.8	7624	1649	1614	22459	12794	13728	2.95	7.76	8.50

[a] Enzymatic depolymerization percentage following a 72 hour incubation in which 6 μg of purified enzyme (S238) PETase, LCC cutinase or Dura PETase) was added to 1 mL of potassium phosphate buffer 50 mM (pH 7.5) with 15 mg of the polymer as substrate. The reaction temperature for S238 A PETase is 30 °C and 70 °C for LCC cutinase and Dura PETase. [b] Initial DPI values of the polymers. [c] Yields of the monomers formed after enzymatic degradation calculated from ^1^H NMR. Concerning the percentage of monomer (HN) formed and for the yield calculation of the monomer from NMR, those peaks that are clearly visible and distinct from the oligomer peaks in the NMR spectra of the polymers after degradation were taken into account. [d] Number average molecular weight after enzymatic degradation. [e] Weight average molecular weight after enzymatic degradation. [f] Polydispersity following enzymatic degradation.

From Figure 2c and Supplementary Figures 1–3, it is clear that the sample contains peaks that are matching with that of the monomer (HN) as well as the polymers. Peaks appearing at ca. 4.5 ppm (−OH), 3.5 ppm, 2.5 ppm, and 1.5 ppm in the NMR spectra of the polyesters after enzymatic degradation correspond to the monomer peaks, and the intensities of the same varied according to the percentage of the diol monomer generated. The remaining peaks of those that correspond to the monomer were merged with the oligomer peaks. For the crystalline polymer (HN_FDCA), the *trans*‐selective PETase S238 A variant showed superior activity, yielding 17 % of the monomers, in contrast to the ca 7 % by Dura and LCC for the same substrate, respectively. The S238 A PETase had a clear preference to the semi‐aromatic FDCA‐derived crystalline substrate, in which the responding polymer chains will preferably adopt extended conformations.[^23^, ^24^, ^25^] The enzymes Dura and LCC, that are selective towards *gauche* PET‐derived substrates,[^20^, ^21^] yielded a higher percentage of monomers for the aliphatic polyesters in which the chains are more flexible and thus prone to adopt twisted conformations. These results aligned with our previous studies that showed that the conformation of the substrate is vital for catalytic activity for PET.^[20]^ The polyester with the longer chain length (HN_DMSc) was preferred by LCC, yielding 10.8 % of monomers and Dura yielded 12.6 %; while S238 A barely reacted, releasing only up to 2 % of the monomers. It is important to note that the biopolyesters did not undergo any pre‐treatment step. Decreasing the molecular weight (*M*_n_, *M*_w_) of the substrates while increasing the surface area can allow the enzymes to more efficiently associate to the polymer resulting in improved hydrolysis. The results summarized in Table 1 suggest other factors beyond the molecular weight of the polymer chain to be of importance in the depolymerization of biopolymers without any further pre‐treatment, even at reaction temperatures far below the materials’ *T*_g_ as manifested by S238 A‐catalyzed depolymerization of HN FDCA (Table 1, top entry).

### Induced Fit Docking of Polyesters to S238 A, Dura and LCC

**Figure 3:**
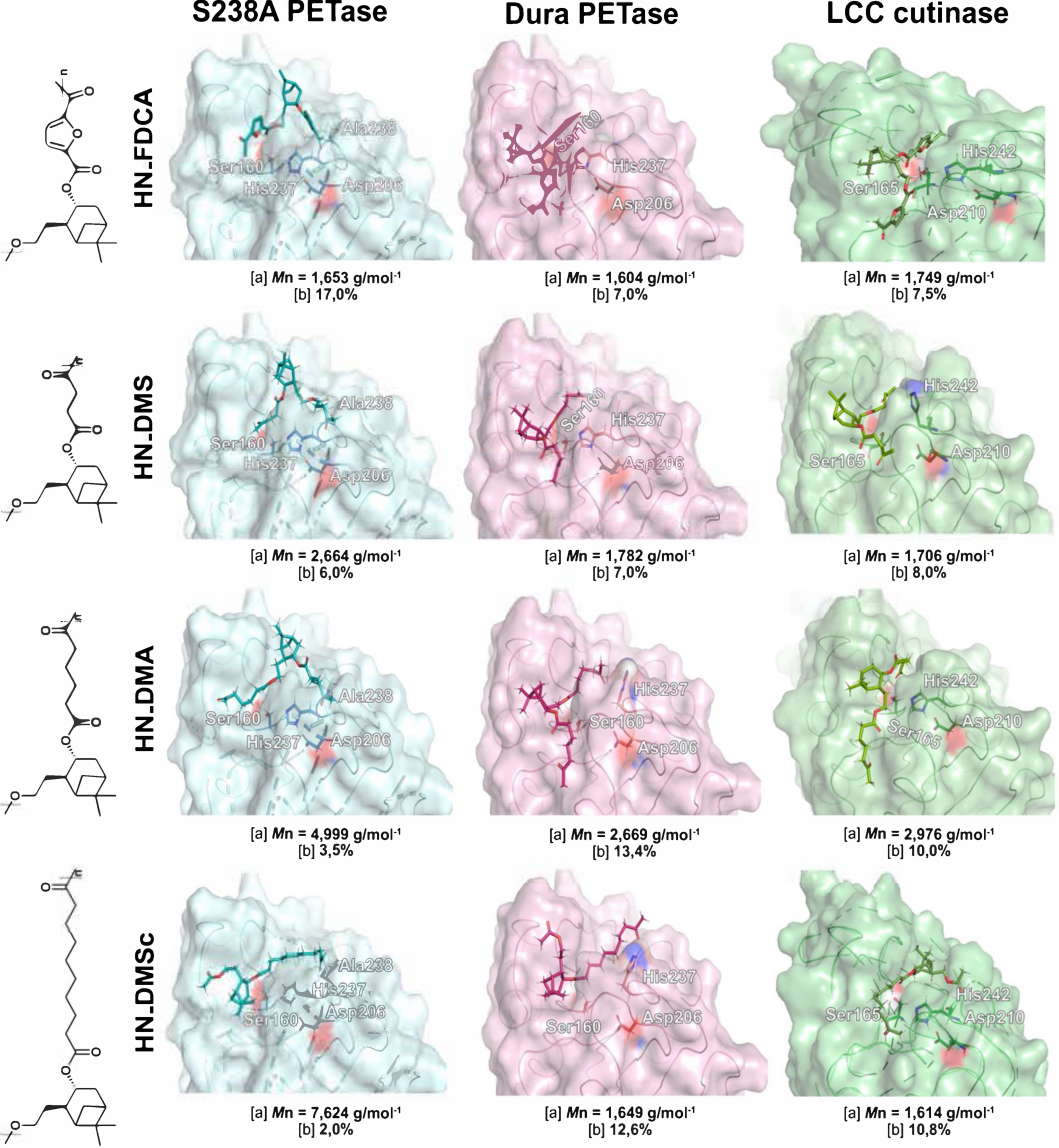
Conformational selection of the S238A *Is*PETase, Dura PETase, and LCC cutinase in which the former prefers straight chains and the latter two twisted substrate conformers. The top‐scoring docking poses of these three enzymes along with the substrates bound to their respective catalytic triads are shown. In each subpanel, [a] refers to the number average molecular weights obtained from SEC following enzymatic degradation, and [b] represents the percentage of the obtained released monomers by each enzyme given for each substrate. The catalytic triad residues are shown and correspond to Ser160, Asp206, His237 for S238 A and Dura *Is*PETases and Ser165, Asp210, and His242 for LCC.

We hypothesized that enzymatic depolymerization efficiency towards the given polyesters would depend not only on the molecular weight and the repeating‐unit carbon chain length but also on their preference towards *trans*‐like and *gauche*‐like chain conformations (schematically shown in Figure 1b, *top*). To better understand the binding ability of the different polyester conformations to the enzymes studied, we performed induced fit docking (IFD) calculations of oligomers (trimers) representing the biopolymers as substrates. We introduced the mutation Ser238Ala in *Is*PETase for the calculations and we have previously seen that this S238 A mutation conferred this enzyme variant a preference towards *trans‐PET‐substrates*,[^20^, ^21^] opposite to the wild‐type or its thermostable analogue Dura. It should be noted that all ligands were prepared using the LigPrep tool in Maestro, resulting in polyesters with retained *anti*‐conformation of the diol oxygens originating from the diol monomer, even though the absolute stereochemistry of the alcohol chain in reference to the cyclobutyl ring in the monomer could change after the structures underwent an energy‐minimization step. After the ligands were prepared, they were used to find the best docking poses for each enzyme. We could observe productive binding of the oligomeric substrates representing the responding biopolymer chain to all the enzymes; manifested by a close‐to Van der Waals nucleophilic attack distance between the Oγ of Ser160 in the PETases and Ser165 in LCC cutinase to the scissile carbonyl carbon (Figure 3, Table 2). The top‐ranked pose of each enzyme‐substrate conformer pair is shown in Figure 3. The corresponding data for the dihedral angle between the diol oxygens of the reacting monomer unit and the distance between the catalytic Ser Oγ and the scissile carbonyl carbon atom for HN_FDCA, HN_DMS, HN_DMA, and HN_DMSc are described in Table 2.

**Table 2:** The dihedral angle between the diol oxygens of the reacting monomer unit for all four biopolymers following IFD analysis with all three biocatalysts. The nucleophilic attack distance (heavy atom) between the catalytic Ser Oγ and the scissile carbonyl atom is also presented in the table. The dihedral angle shown in this table refers to *gauche‐*like if the absolute value of the angle is between 0° and 90° whereas it is considered *trans‐*like if the substrate chain has an angle between 90° and 180°.

**Polyester**	**Dihedral angles (°)**			**Nucleophilic distance (Å)**		
	**S2388 A**	**Dura**	**LCC**	**S238 A**	**Dura**	**LCC**
**HN FDCA**	−161.3	−86.3	89.9	3.5	3.4	4.1
**HN DMS**	148.9	−37.2	−7.8	5.2	3.2	4.4
**HN DMA**	123.8	−36.7	−41.9	4.1	4.3	3.4
**HN DMSc**	−151.9	93.1	58.6	4.2	4.7	4.6

The synthesized biopolymers showed different physical properties, the HN_FDCA polyester is highly crystalline while HN_DMS, HN_DMA, and HN_DMSc are amorphous. Based on the results obtained from the IFD analysis, we were able to better understand the structure‐functional relationship between the enzymes and these biopolymers. It is clear that the S238 A PETase is more active in the degradation of the crystalline polyester HN FDCA, whereas Dura PETase and LCC cutinase are more active in the degradation of the amorphous polyesters. In fact, Dura PETase and LCC cutinase show a similar degree of activity for depolymerization of the amorphous polyesters HN_DMS, HN_DMA, HN_DMSc that have a varying chain length (see Table 1). Interestingly, from Table 2 it is evident that the dihedral angles of the oligomers of the top‐scoring docking poses exclusively represent the straight‐chain *trans‐like* conformation for S238 A for all of the substrates. In contrast, Dura and LCC show preference towards twisted chain *gauche‐*like polymer conformations that are abundant in amorphous structures where the chains are mobile.

**Figure 4:**
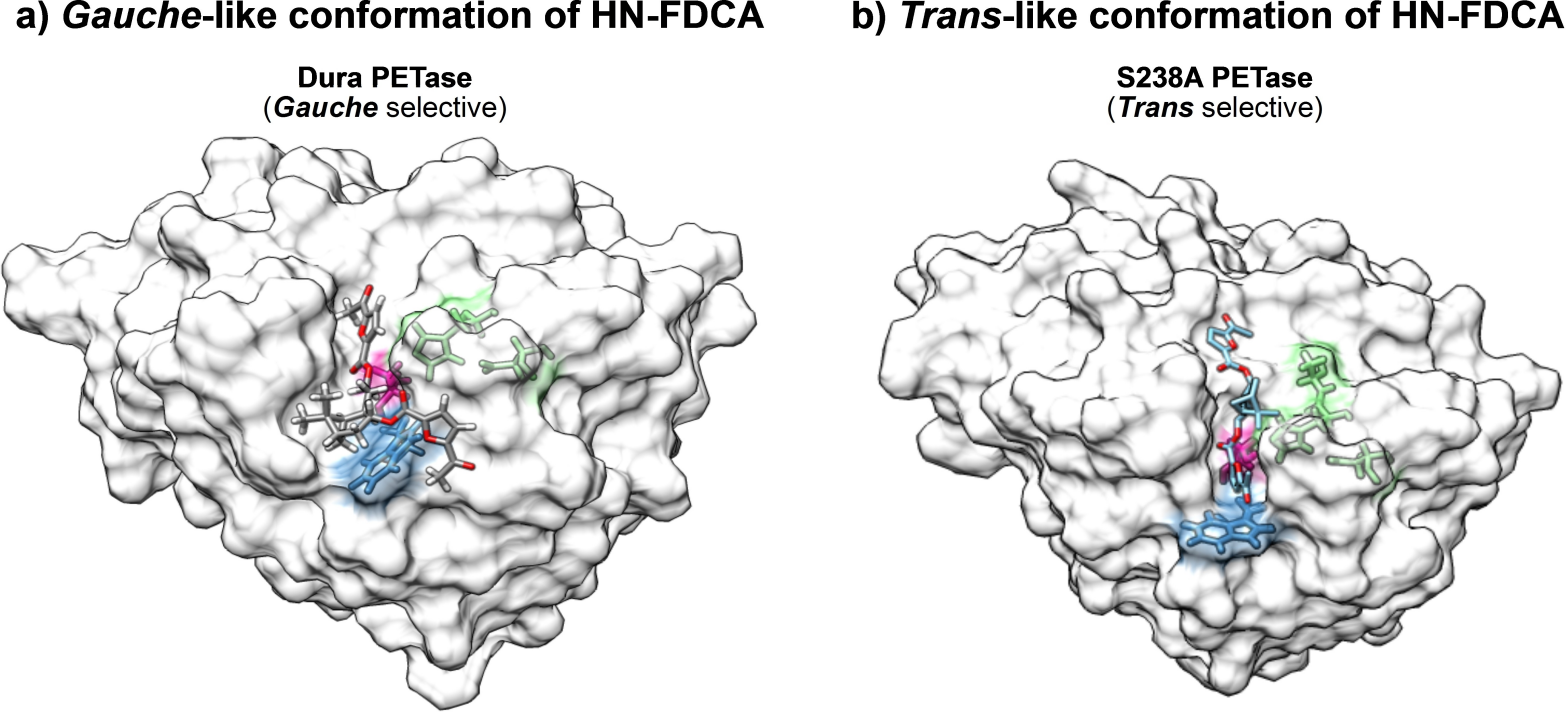
Conformational selection for biopolymer degradation. a) The enzyme Dura PETase is shown with the HN FDCA substrate docked into its active site. The “wobbling” residue W185 (blue) is positioned in a way that does not allow the biopolymer to bind as a straight chain, causing the substrate to acquire a *gauche*‐like conformation when bound to the active site (parts shown in green). The catalytic S160 is highlighted in bright pink. b) The enzyme S238 A PETase is shown with the HN FDCA substrate docked into its active site. The “wobbling” residue W185 (blue) adopts a flat (perpendicular) orientation which allows for the biopolymer to bind in a straight chain when complexed to the active site (part shown in green). The catalytic S160 is highlighted in bright pink.

Interestingly, from the docking results we were able to see that the wobbling residue W185^[26]^ in the *Is*PETase variants seems to act as a gate to dictate in which conformation the biopolymer substrate will bind.^[27]^ As shown in Figure 4, the Dura PETase, W185 is in a perpendicular orientation blocking the polyester to bind in a straight chain, enforcing the chain to adopt a *gauche*‐like twisted conformation. Conversely, the S238 A PETase variant W185 is flat allowing the chain to bind to the active site as a straight chain. The enzymes’ activity towards the different biopolymers seems to rely highly on conformations adopted by the polymeric chains in amorphous and crystalline regions and their different affinity to enzyme active sites. For the more crystalline polymer (HN_FDCA), the packed extended conformation is preferred; one that suitably binds to S238 A PETase whereas Dura PETase and LCC cutinase prefer amorphous substrates with more mobile chains.

## Conclusions

There has not been enough time for enzymes to develop to degrade man‐made polyesters efficiently, including synthetic polymers from renewable resources. However, by understanding the fundamental structure‐function relationship between an enzyme and its substrate, optimizing the depolymerization activity towards bio‐based polyesters could be facilitated. We have previously shown the importance of matching discrete polymer geometries to enzyme ground‐state conformations for efficient hydrolysis of PET.^[20]^ Herein, we have studied the depolymerization of the novel set of terpene‐derived biopolymers HN_FDCA, HN_DMS, HN_DMA, and HN_DMSc by the S238 A PETase, Dura PETase, and LCC cutinase and our data suggests a general importance of conformational selection that extends beyond PET. According to this model, the basal propensity of a polymer chain to adopt either an extended straight conformation or twisted chain and their responding affinities to enzyme active sites should be considered in enzymatic degradation, alongside already established parameters including molecular weight, degree of crystallinity, surface hydrophilicity, and water absorption.[^28^, ^29^, ^30^]

## Experimental Section

### Synthesis of Monomer *trans*‐Hydroxy nopol (HN)

The monomer was synthesized by following our previously published synthetic strategy^[22]^ which involves the hydroboration of the nopol by borane‐THF followed by oxidation using H_2_O_2_. For the detailed procedure refer to our previous work.^[22]^

### Synthesis of Polyesters (HN FDCA, HN DMS, HN DMA, and HN DMSc) from *trans*‐Hydroxy nopol (HN)

The polyesters were synthesized by following our previously published synthetic protocol.^[25]^ The polyesters were synthesized by transesterification followed by polycondensation with a series of aromatic (2,5‐dimethyl furan dicarboxylate, and aliphatic diesters (dimethyl succinate, dimethyl adipate, dimethyl sebacate) using DBTO as a catalyst at 120–140 °C. For a detailed synthetic procedure, refer to our previous work.^[21]^

### Expression of S238 A and Dura PETase and LCC Cutinase

All the genes for the corresponding enzymes were subcloned into PET‐21b (+) plasmids and transformed into *Escherichia coli* CD43 cells that were grown on Nutrient Agar plates with 100 μg mL^−1^ of ampicillin (Sigma Aldrich, USA). The proteins were expressed and produced the following way: The cells transformed with the enzyme plasmids were precultured in 2YT media (16 g/L tryptone, 10 g/L yeast extract, and 5 g/L NaCl in MiliQ water) with 100 μg mL^−1^ of ampicillin overnight. The next day, the cultures were inoculated into 200 mL of 2YT and grown at 37 °C under shaking at 200 rpm until an OD_600_ of 0.6 was reached. Once the desired OD_600_ was reached, 1 mM of isopropyl *β*‐d‐1‐thiogalactopyranoside (IPTG) was added and the cells were incubated for 20 hours at 20 °C 160 rpm. After the incubation time was finished, the cell pellets were harvested by centrifugation at 4,800×g for 20 minutes.

### Purification of S238 A‐ and Dura‐ PETase and LCC Cutinase

The cells were lysed to obtain the expressed proteins by B‐PER complete lysis buffer containing 20 mM imidazole at 25 °C 80 rpm for 20 minutes. After the lysis of the cells, the supernatant containing the crude enzyme was collected after centrifugation. To purify the enzymes, an ÄKTA Start (GE Healthcare, Sweden) instrument was used. The HisTrap FF column was equilibrated with washing buffer (10 mM sodium phosphate, 500 mM NaCl, and 40 mM imidazole) and the enzyme was eluted with an imidazole gradient (from 40 mM to 300 mM) in the same buffer at a flow rate of 1 ml/min. Finally, the enzymes were desalted using a PD‐10 column into 10 mM Tris‐HCl buffer pH 7.5. Proteins were confirmed by Sodium dodecyl sulphate‐polyacrylamide (SDS‐Page) gel electrophoresis with a gradient of 4–15 % (Mini‐Protean TGX Stain‐Free, BioRad, Sweden). The SeeBlueTM Plus2 (Thermo Fisher Scientific, USA) pre‐stained protein standard was used as a marker.

### Enzymatic Degradation of Polymers

The polymers HN DMS, HN DMA, HN DMSc, and HN FDCA were subjected to enzymatic polymerization. For each reaction, 6 μg of purified enzyme (S238 A PETase, LCC cutinase or Dura PETase) were added to 1 mL of potassium phosphate buffer 50 mM (pH 7.5) with 15 mg of polymer as substrate. The polymeric substrate was not subjected to any pre‐treatment. The samples were incubated for 72 hours at 30 °C for the reaction with the S238 A PETase and 70 °C for the reactions with LCC cutinase and Dura PETase. Finally, the resulting products from the depolymerization reaction were freeze‐dried using a Heto LyoLab 3000.

### Proton Nuclear Magnetic Resonance (^1^H NMR)

^1^H NMR spectra were recorded using a Bruker Avance AM 400 NMR instrument using CDCl_3_ as solvent. The polymers were solubilized and submitted for proton analysis with 128 scans.

### Size Exclusion Chromatography (SEC)

SEC elugrams were obtained on a Malvern GPCMAX instrument equipped with PLgel 5 μm guard column (7.5×50 mm) and two PLgel 5 μm MIXED−D (300×7.5 mm) analysis columns kept at 35 °C. M_n_ and Ð values are reported versus a calibration of narrow disperse polystyrene standards. The eluent consisted of HPLC grade CHCl_3_ containing 2 % (v/v) toluene. Polyesters were dissolved in SEC eluent and kept for 12 h. Afterward, the samples from the enzymatic degradation were filtered using a membrane filter and transferred into a special SEC vial, and a volume of 100 μL of each sample was injected.

### Induced Fit Docking (IFD)

X‐ray crystal structures of IsPETase (PDB ID: 6EQE^[13]^), Dura PETase (PDB ID: 6KY5^[16]^) and LCC–Cutinase (PDB ID: 4EB0^[31]^) were downloaded from the RCSB Protein Data Bank. All protein structures were prepared for IFD docking using the protein preparation wizard in the software Schrödinger Maestro Suite (Schrödinger, LCC, USA). For the Dura PETase, we retained only chain A for the IFD calculations. All crystallographic water molecules were removed, and the missing hydrogens were added at a set pH of 7.5. The polyester substrates were prepared in Maestro using the LigPrep wizard and were all energy‐minimized using the OPLS4 force field. Finally, all four of the polyesters were used to generate IFD calculations with all three of the enzymes.

## Conflict of Interests

POS and GR have submitted a patent application on the materials under study to the Swedish patent office (#2430256‐4)

1

## Supporting information

As a service to our authors and readers, this journal provides supporting information supplied by the authors. Such materials are peer reviewed and may be re‐organized for online delivery, but are not copy‐edited or typeset. Technical support issues arising from supporting information (other than missing files) should be addressed to the authors.

Supporting Information

## Data Availability

The data that support the findings of this study are available from the corresponding author upon reasonable request.
