# Supplementary material for: Conformational Selection in Enzyme‐Catalyzed Depolymerization of Bio‐based Polyesters
Source: Chembiochem. 2024 Sep 17;26(2):e202400456. doi: 10.1002/cbic.202400456 (PMC11776369; doi:10.1002/cbic.202400456)
Supplement: Supplementary file 1 — Supporting Information [file CBIC-26-e202400456-s001.pdf]

ChemBioChem

Supporting Information

Conformational Selection in Enzyme-Catalyzed Depolymerization of Bio-based Polyesters

Ximena Lopez-Lorenzo, Ganapathy Ranjani, and Per-Olof Syrén*

Conformational Selection in Enzyme-Catalyzed Depolymerization of Bio-based Polyesters

Ximena Lopez-Lorenzo[a,b], Ganapathy Ranjani [a,b] and Per-Olof Syrén[a,b]

[a] School of Engineering Sciences in Chemistry, Biotechnology and Health, Department of Fibre and Polymer Technology, KTH Royal Institute of Technology, Stockholm, Sweden.
[b] School of Engineering Sciences in Chemistry, Biotechnology and Health, Science for Life Laboratory, KTH Royal Institute of Technology, Stockholm, Sweden.

Supporting Information

Abstract: Enzymatic degradation of polymers holds promise for advancing towards a bio-based economy. However, the bulky nature of polymers presents challenges in accessibility for biocatalysts, hindering depolymerization reactions. Beyond the impact of crystallinity, polymer chains can reside in different conformations affecting binding efficiency to the enzyme active site. We previously showed that the *gauche* and *trans* chain conformers associated with crystalline and amorphous regions of the synthetic polyethylene terephthalate (PET) display different affinity to PETase, thus affecting the depolymerization rate. However, structural-function relationships for biopolymers remain poorly understood in biocatalysis. In this study, we explored biodegradation of previously synthesized bio-polyesters made from a rigid bicyclic chiral terpene-based diol and copolymerized with various renewable diesters. Herein, four of those polyesters spanning from semi-aromatic to aliphatic were subjected to enzymatic degradations in concert with induced-fit docking (IFD) analyses. The monomer yield following enzymatic depolymerization by *Is*PETase S238A, Dura and LCC ranged from 2%-17% without any further pre-treatment step. The degradation efficiency was found to correlate with the extent of matched substrate and enzyme conformations revealed by IFD, regardless of the actual reaction temperature employed. Our findings demonstrate the importance of conformational selection in enzymatic depolymerization of biopolymers. A straight or twisted conformation of the polymer chain is crucial in biocatalytic degradation by showing different affinities to enzyme ground-state conformers. This work highlights the importance of considering the conformational match between the polymer and the enzyme to optimize the biocatalytic degradation efficiency of biopolymers, providing valuable insights for the development of sustainable bioprocesses.

Table of Contents **Page**

1. NMR Spectra of the polyesters after enzymatic degradation

   - Comparison of ^{1}H NMR spectra of polyester HN_DMS before and after enzymatic degradation 3
   - Comparison of ^{1}H NMR spectra of polyester HN_DMA before and after enzymatic degradation 4
   - Comparison of ^{1}H NMR spectra of polyester HN_DMSc before and after enzymatic degradation 5

2. SDS-Page gel for protein expression 6

3. Author contributions 6

1. **NMR Spectra of the polyesters after enzymatic degradation:**

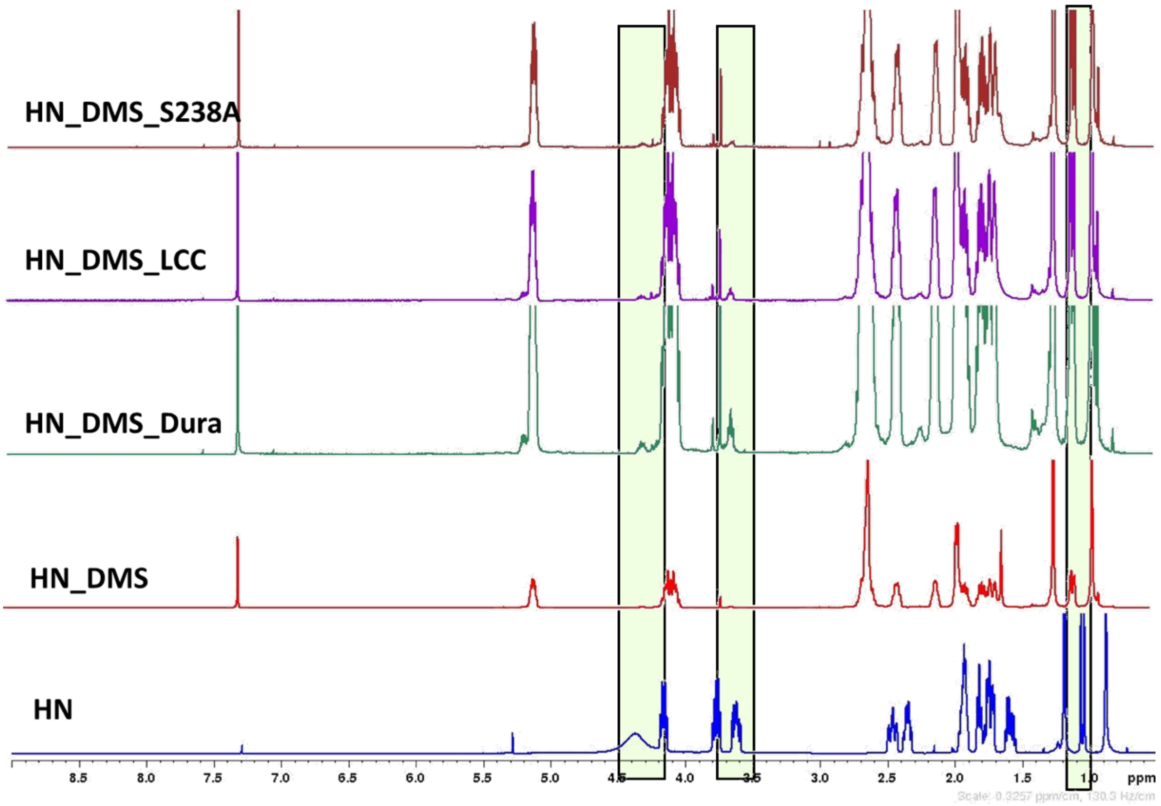

Figure S1. Comparison of NMR spectra of polyester **HN_DMS** before and after enzymatic degradation

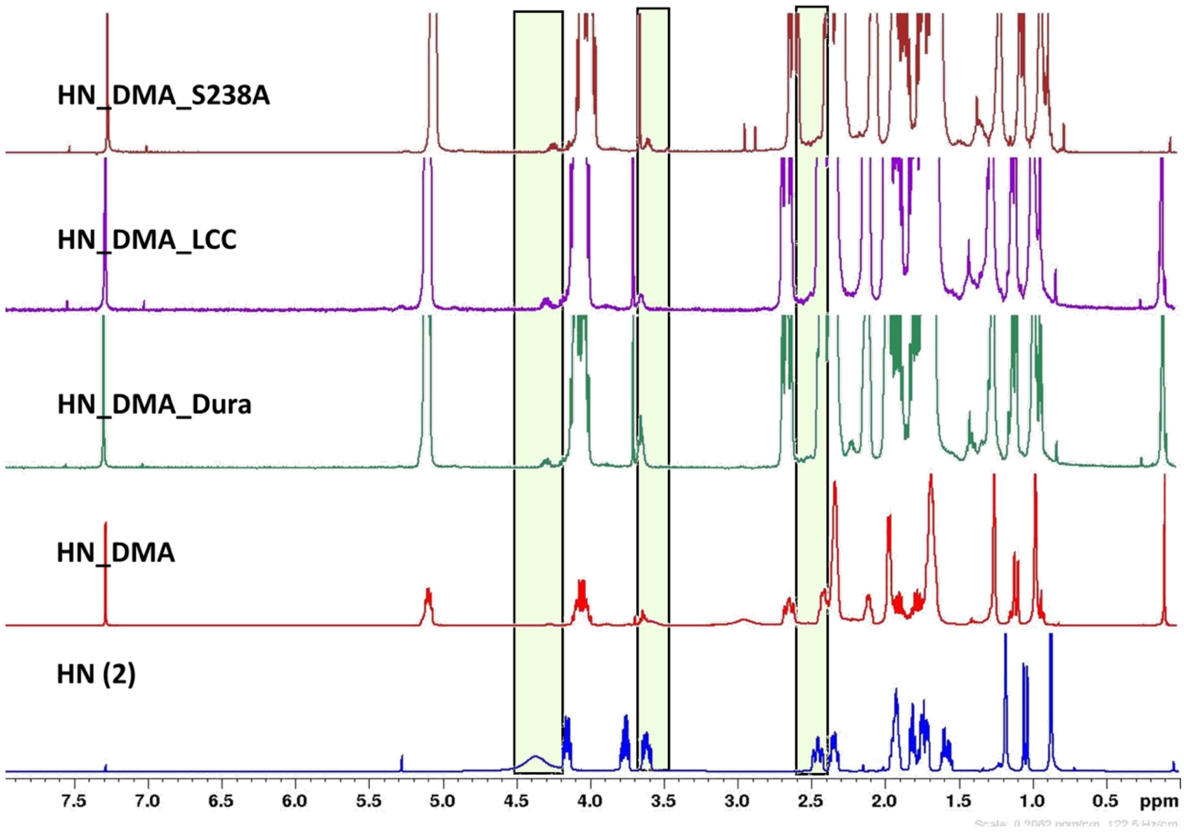

Figure S2. Comparison of NMR spectra of polyester **HN_DMA** before and after enzymatic degradation

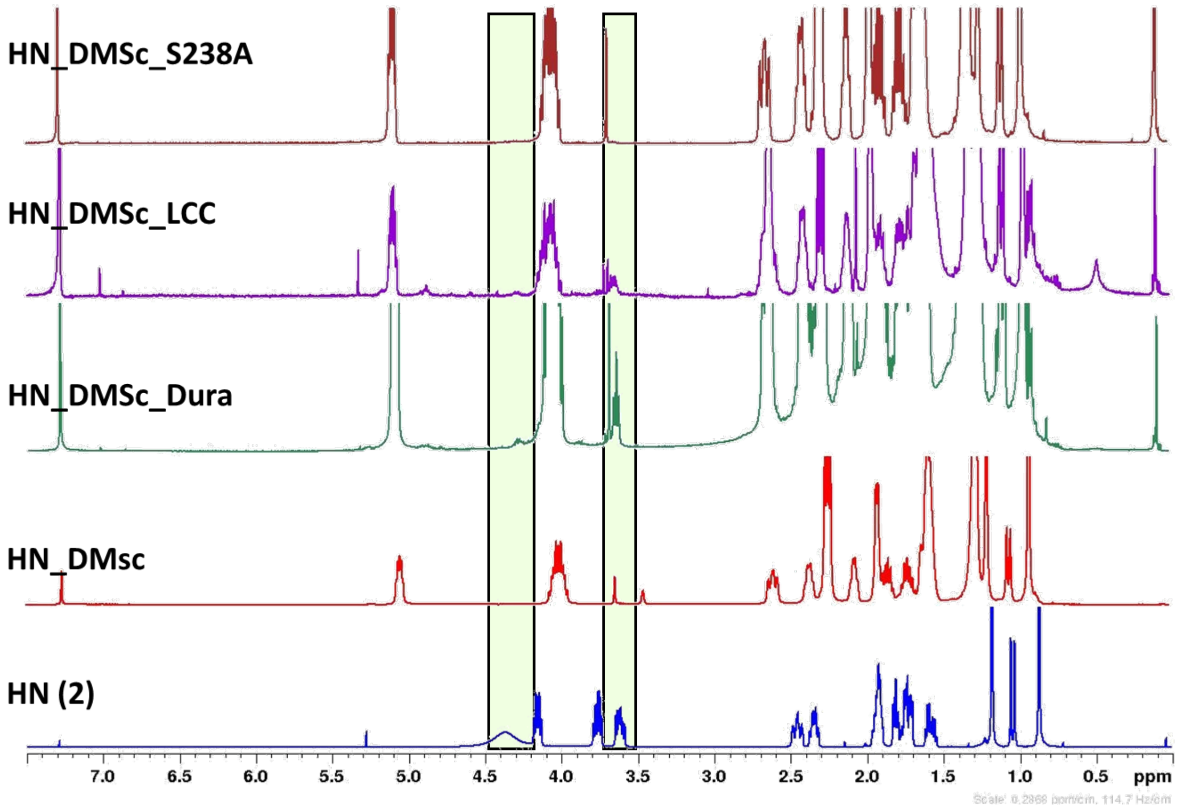

Figure S3. Comparison of NMR spectra of polyester **HN_DMSc** before and after enzymatic degradation

2. **SDS-Page gel for protein expression:**

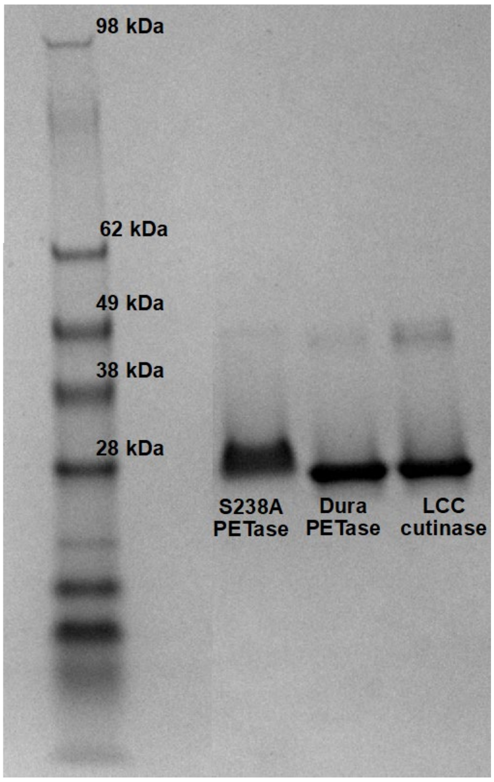

Figure S4. SDS-Page Gel of S238A PETase, LCC cutinase and Dura PETase

Author contributions:

P.O.S and X.L.L designed the research; X.L.L performed IFD analysis; X.L.L and R.G performed the experiments. R. G collected NMR, SEC data, analyzed, plotted them, and helped in revising the manuscript. X.L.L prepared the manuscript; P.O.S supervised the research and revised the manuscript. All authors approved the final version of the manuscript.
